# Calcium-assisted sortase A cleavage of SUMOylated metallothionein constructs leads to high-yield production of human MT3

**DOI:** 10.1186/s12934-023-02134-x

**Published:** 2023-07-11

**Authors:** Avinash Kumar Singh, Artur Krężel

**Affiliations:** Department of Chemical Biology, Faculty of Biotechnology, University of Wrocław, Joliot-Curie 14a, 50-383 Wrocław, Poland

**Keywords:** Metallothionein, Sortase, SUMO, Ulp1, Recombinant expression, Plasmid generation, IMAC, Protein purification

## Abstract

**Background:**

Mammalian metallothioneins (MTs) are small (6–7 kDa), intracellular, cysteine-rich, metal-binding proteins involved, inter alia, in the homeostasis of zinc and copper, detoxification of heavy metals, antioxidation against reactive oxygen species, and protection against DNA damage. The high cysteine content (~ 30%) in MTs makes them toxic to bacterial cells during protein production, resulting in low yield. To address this issue, we present for the first time a combinatorial approach using the small ubiquitin-like modifier (SUMO) and/or sortase as fusion tags for high-level expression of human MT3 in *E. coli* and its purification by three different strategies.

**Results:**

Three different plasmids were generated using SUMO, sortase A pentamutant (eSrtA), and sortase recognition motif (LPETG) as removable fusion tags for high-level expression and purification of human MT3 from the bacterial system. In the first strategy, SUMOylated MT3 was expressed and purified using Ulp1-mediated cleavage. In the second strategy, SUMOylated MT3 with a sortase recognition motif at the N-terminus of MT3 was expressed and purified using sortase-mediated cleavage. In the final strategy, the fusion protein His_6_-SUMO-eSrtA-LPETG-MT3 was expressed and purified by one-step sortase-mediated inducible on-bead autocleavage. Using these three strategies the apo-MT3 was purified in a yield of 11.5, 11, and 10.8 mg/L, respectively, which is the highest yield achieved for MT expression and purification to date. No effect of MT3 on Ni^2+^-containing resin was observed.

**Conclusion:**

The SUMO/sortase-based strategy used as the production system for MT3 resulted in a very high expression level and protein production yield. The apo-MT3 purified by this strategy contained an additional glycine residue and had similar metal binding properties as WT-MT3. This SUMO-sortase fusion system is a simple, robust, and inexpensive one-step purification approach for various MTs as well as other toxic proteins with very high yield via immobilized metal affinity chromatography (IMAC).

**Supplementary Information:**

The online version contains supplementary material available at 10.1186/s12934-023-02134-x.

## Background

Mammalian metallothioneins (MTs) are a family of small (6–7 kDa), intracellular, cysteine-rich metal-binding proteins [1]. Initially, MT was isolated from the equine kidney cortex as a Cd^2+^-binding protein in 1957 [2]. It is reportedly involved in a variety of physiological functions including homeostasis of essential metals, such as Zn^2+^ and Cu^+^, radical scavenging, oxidative stress protection, etc., and toxic-metal detoxification [1, 3–5]. In humans, there are four major isoforms of MTs (MT1-MT4), among which MT1 is present in numerous sub-isoforms (MT1a-MT1x) [1, 6]. They demonstrate different metal-binding properties and tissue localization such as cytosol, nucleus, mitochondria, and the extracellular environments [7–9]. MT1 and MT2 consist of 61 amino acid residues and are ubiquitously expressed to maintain mainly Zn^2+^ cellular homeostasis [10]. MT3 is the longest among human isoforms with a length of 68 amino acids and is predominantly expressed in hippocampal neurons and astrocytes of the brain [11, 12]. It was recognized as a Zn^2+^- but also Cu^+^-binding protein – being more specific for the latter and binding it tightly [12, 13]. The expression of MT3 in the brain is affected by a range of other neurological diseases as well as by brain inflammation and injury. MT4 is 62 residues long and is only expressed in highly specialized tissues such as squamous differentiated epithelia, which are found in the skin, tongue, and esophagus [14]. MTs are dumbbell-shaped proteins having 20 cysteine residues distributed in two thiol-rich domains, namely β and α, containing M_3_Cys_9_ and M_4_Cys_11_ (M denotes divalent metal ion) clusters, respectively, separated by a conserved tripeptide LysLysSer (KKS) linker [1, 15, 16].

Although metallothioneins were for many years purified from animal tissues, mostly the liver in the case of mammals, currently the most common technique for obtaining particular homogenous MT isoforms is the production of recombinant protein directly or in the form of a fusion protein from *E. coli* [17–20]. However, the low stability and cell toxicity of high thiol content in MT hinders its high expression in *E. coli* [21–25]. Various fusion tags such as ubiquitin (Ub), glutathione S-transferase (GST), S-tag, maltose binding protein (MBP), etc., have been shown to enhance protein stability by acting as chaperones, and frequently the fusion protein is expressed as a soluble protein rather than in inclusion bodies. Among these fusion tags, GST and S-tag have achieved some success in the purification of mammalian MTs [24–27]. Although the use of these tags resulted in improved yield, they have certain limitations. The removal of both tags from the expressed fusion protein usually leaves a stretch of additional amino acid residues. Since MTs are small proteins, addition of a stretch of amino acid residues to the final protein may interfere with its metalation (metal binding process) and biochemical properties. Another widely used method for purifying mammalian MTs is intein-mediated purification with an affinity chitin-binding tag (IMPACT) system, in which MT is expressed as an intein-fusion protein and the intein tag is proteolytically cleaved by incubating it with D,L-dithiothreitol (DTT) [28, 29]. Application of the IMPACT system increased the expression of thionein, and the final protein lacked additional amino acid residues. Unfortunately, this system is time-consuming, as during elution the fusion protein needs to be incubated for 48 h with DTT for cleavage [28]. Also, the chitin resin used during purification is expensive and is used in large quantities, which increases the cost of production. Expression and purification of tag-less MTs have also been reported by some research groups [30]. In this tag-less purification method, the cDNA coding for the MT without any tag or surplus residues is cloned and expressed from an expression vector followed by its purifications using size-exclusion and anion-exchange chromatography [30–32]. Although this tag-less purification system is quite efficient, it is time-consuming as it requires ethanol precipitation and several chromatography steps including size exclusion as well as ion exchange. A significant amount of the protein is lost during these numerous purification steps.

Lately, the use of SUMO/Ub as a fusion tag in recombinant protein expression has been increasing and it has been successfully used to express plant ligases in bacteria as soluble and active forms [33–37]. The anti-proteolytic property of the SUMO tag significantly increases the expression levels, correctly facilitates target protein folding, and increases the solubility of the recombinant proteins [38–42]. It is especially important for MTs since they are prone to proteolysis and their fusion to the SUMO tag more likely increases production yield [43, 44]. After purification of the fusion protein, the SUMO tag can be efficiently removed using SUMO-specific proteases. SUMO in combination with the GST tag has been used to express and purify modified MT in high yields in *E. coli.* [45]*.* The purified GST-SUMO-MT was then administered in mice for in vivo studies and was able to effectively protect multiple organs against aging, diabetes, and ionizing radiation-related oxidative damage [45]. Another protein fusion system using the sortase tag has been developed to purify tag-less recombinant proteins in a single step by inducing self-cleavage [46]. This fusion protein consisted of three components: an N-terminal His_6_-tag, Δ59 *Staphylococcus aureus* sortase A (SaSrtA), and an LPETG linker followed by the protein of interest at the C-terminus. After immobilizing the fusion protein on Ni^2+^-NTA beads, on-column SaSrtA-mediated self-cleavage in the presence of Ca^2+^ is done by cleaving the Thr-Gly peptide bond of the sortase recognition motif Leu-Pro-Glu-Thr-Gly (LPETG). A protein having an extra glycine residue at its N-terminus is released while the N-terminal portion of the fusion protein remains bound to the Ni^2+^-NTA beads. It has also been reported that sortase can successfully cleave and dissociate *Synechococcus elongatus* metallothionein from the Lpp′-ompA-mt-ChBD carrier protein displayed on the *E. coli* cell surface [47].

The present study aimed to develop an efficient expression and Ca^2+^-inducible sortase-mediated on-bead self-cleavage purification strategy for increasing the overall yield of human MT3 in *E. coli* using SUMO and/or sortase as fusion tags. The recombinant fusion proteins (His_6_-SUMO-MT3, His_6_-SUMO-LPETG-MT3, and His_6_-SUMO-eSrtA-LPETG-MT3) were expressed as soluble proteins in very high yield in *E. coli* and were purified using Ni^2+^-NTA immobilized metal affinity chromatography (IMAC). The final metallothionein-3 obtained after eSrtA cleavage contains just one additional glycine residue at its N-terminus (G-MT3) and its physiochemical properties were found to be very similar to those of native sequence human WT MT3 purified using the IMPACT system. Using this strategy MT3 was obtained in very high yield, reportedly the highest of the available strategies. Moreover, our study also showed that the Ni^2+^-NTA-based purification system does not influence the metal content of recombinant MT3 and vice versa.

## Material and methods

### Reagents

Acetonitrile (MeCN), HCl (trace metal grade), NaClO_4_ H_2_O, ZnSO_4_·7H_2_O, 4-(2-pyridylazo)resorcinol (PAR), and Triton X-100 were from Merck Millipore. Tris(2-carboxyethyl)phosphine hydrochloride (TCEP), tryptone, yeast extract, LB Broth, agar, agarose, isopropyl-β-D-1-thiogalactopyranoside (IPTG) and sodium dodecyl sulfate (SDS) were purchased from Lab Empire. Ampicillin, chloramphenicol, 1,4-dithiothreitol (DTT) and Tris base were from Roth, pTYB21 vector and chitin resin from New England BioLabs, Chelex 100 resin from Bio-Rad, 4-(2-hydroxyethyl)piperazine-1-ethanesulfonic acid sodium salt (HEPES), 5,5ʹ-dithiobis-(2-nitrobenzoic acid) (DTNB) from TCI Europe. To eliminate trace metal ion contamination all pH buffers were treated with Chelex 100 resin (Bio-Rad) and degassed over 2 h prior to use. For the culture of *E. coli*, Luria–Bertani (LB) medium and agar plates were used.

### DNA Manipulation

All primers and plasmids used in this study are listed in Additional file 1: Table S2. MT3-pTYB21 plasmid (Addgene plasmid #105710) was used as a template for the generation of LPETG-MT3 using appropriate primers [48]. Sortase A pentamutant (eSrtA) in pET29 was a gift from David Liu (Addgene plasmid # 75144) [49]. The plasmids pBHRSF184 (Addgene plasmid # 89482) and pFGET19_Ulp1 (Addgene plasmid # 64697) were a gift from Hideo Iwai [33]. pETM11-SUMO-SNCA-GFP (Addgene plasmid # 107292) was a gift from Dmytro Yushchenko [50]. The plasmids were isolated using the miniprep plasmid isolation protocol and their correctness was verified by DNA sequencing. For a larger amount of the plasmid, plasmid DNA was isolated from 50 ml of overnight cultures using the Qiagen Hi-Speed Plasmid Purification Kit (Qiagen, Germany). All PCR reactions were performed with the Fast Start High Fidelity kit (Roche, Germany), according to the manufacturer’s directions, using PTC-150 Mini-Cycler (MJ Research). Following an initial 2 min denaturation step at 95 °C, 35 cycles of 30 s at 95 °C, 30 s at 57 °C, and 1–2 min at 72 °C were performed depending on the length of the target sequence. The amplification was terminated with 2 min incubation at 72 °C. The PCR products were run on 1% gel (w/v) agarose gels and purified with the QIAquick PCR purification kit (Qiagen GMBH, Germany) before cloning. *E. coli* DH5α cells were transformed with ligated plasmids according to the CaCl_2_ method.

### Construction of SUMO-MT3 plasmid

PCR amplification was used to amplify a DNA fragment containing the coding sequence of MT3 from the MT3-pTYB21 plasmid template (primers are listed in Additional file 1: Table S2). The PCR product was double digested with BamHI-HindIII restriction enzymes and was ligated into the same sites of the plasmid pBHRSF184 to make the SUMO-MT3 plasmid. This plasmid is now deposited in Addgene under ID #200303.

### Construction of SUMO-LPETG-MT3 plasmid

PCR amplification was used to amplify a DNA fragment containing the coding sequence of LPETG fused to the coding sequence of MT3 from the MT3-pTYB21 plasmid template (primers are listed in Additional file 1: Table S2). The PCR product was double digested with BamHI-HindIII restriction enzymes and was ligated into the same sites of the plasmid pBHRSF184 to make the SUMO-LPETG-MT3 plasmid. This plasmid is now deposited in Addgene under ID #200304.

### Construction of eSrtA-MT3 plasmid

This plasmid was generated from pETM11-SUMO-SNCA-GFP in a two-step process. In the first step, the eSrtA gene was PCR amplified from the eSrtA-pET29 plasmid, double digested with BamHI and NdeI restriction enzymes, and was ligated into the same sites of the plasmid pETM11-SUMO-SNCA-GFP to make the pETM11-SUMO-eSrtA-GFP plasmid. In the next step, PCR-amplified LPETG-MT3 was double digested with NdeI and XhoI restriction enzymes and was ligated into the same sites of the plasmid pETM11-SUMO-eSrtA-GFP to make the eSrtA-MT3 plasmid having the sequence pETM11-SUMO-eSrtA-LPETG-MT3. This plasmid is now deposited in Addgene under ID #200305. A control plasmid encoding gene construct for His_6_-eSrtA-LPETG-MT3 (eSrtA-MT3 without SUMO) fusion protein was also generated by cloning eSrtA-LPETG-MT3 gene between BamHI and XhoI sites of a pET28-TCT universal plasmid (data not shown). The information about this control fusion protein's amino acid sequence and mass can be found in (Additional file 1: Fig. S4 and Additional file 1: Table S1).

### Expression and purification of eSrtA, Ulp1, MT2-WT, and MT3-WT

The eSrtA and Ulp1 enzymes were expressed and purified using previously described methods [49, 51, 52]. MT2-WT and MT3-WT were expressed and purified using a previously established protocol using MT2-pTYB21 (Addgene plasmid #105693) and MT3-pTYB21 plasmids (Addgene plasmid #105710) from our laboratory [48, 53].

### Expression and purification of SUMO-MT3, SUMO-LPETG-MT3, and eSrtA-MT3

For protein expression, the positive clones were transformed into BL21 (DE3) RIL *E. coli* cells and a primary culture was inoculated in LB medium at 37 °C overnight with constant shaking at 180 rpm. Secondary culture was inoculated in 1L LB medium by adding 1% (v/v) primary culture and grown at 37 °C until OD_600_ reached 0.6–0.8. Cells were induced with 0.1 mM IPTG and incubated overnight at 20 °C with constant shaking at 120 rpm. The cell pellet was collected by centrifugation (4,000 × *g* for 10 min, 4 °C), resuspended in 50 ml of ice-cold lysis buffer (20 mM HEPES pH 8.0, 500 mM NaCl, 1 mM TCEP, 0.1% tritonX-100, 10 mM imidazole), and sonicated for 20 min (5 s “on” and 5 s “off”) followed by centrifugation (16,000 × *g* for 45 min, 4 °C) [53]. The clear supernatant was incubated for 2 h with Ni^2+^-NTA resin (pre-equilibrated in lysis buffer) for protein binding. The resin was then washed 4–5 times with wash buffer (20 mM HEPES pH 8.0, 500 mM NaCl, 1 mM TCEP, 50 mM imidazole). SUMO-MT3 and SUMO-LPETGM-MT3 fusion proteins were eluted in elution buffer (20 mM HEPES pH 8.0, 150 mM NaCl, 1 mM TCEP, 350 mM imidazole) and concentrated using Amicon Ultra-4 Centrifugal Filter Units with a membrane cut-off of 10 kDa (Merck Millipore, USA). The imidazole was then removed from the eluted protein by size-exclusion chromatography on a PD-10 desalting column using calcium-deficient sortase reaction buffer (150 mM NaCl, 50 mM Tris pH 7.5, and 5 mM DTT).

### Cleavage of the fusion protein SUMO-MT3 with Ulp1

The desalted fusion protein SUMO-MT3 was digested at 37 °C for 4 h by the addition of a final concentration of 37 µM Ulp1 protease [33]. The His_6_-SUMO and Ulp1-His_6_ were successfully separated from the pure GS-MT3 by a second Ni^2+^-NTA purification. The GS-MT3 obtained after Ulp1 cleavage contained two additional amino acid residues: Gly and Ser. The purified and cleaved GS-MT3 was collected and concentrated.

### Cleavage of the fusion protein SUMO-LPETG-MT3 with eSrtA

The desalted fusion protein SUMO-LPETG-MT3 was digested at 37 °C for 4 h by the addition of a final concentration of 100 µM eSrtA and 5 mM CaCl_2_. The His_6_-SUMO and eSrtA-His_6_ were successfully separated from the pure G-MT3 by a second Ni^2+^-NTA purification. The purified and cleaved G-MT3 (containing one additional Gly residue) was collected and concentrated.

### On-bead cleavage and purification of the fusion protein eSrtA-MT3

Ni^2+^-NTA resin bound to eSrtA-MT3 was equilibrated with calcium-deficient sortase reaction buffer (150 mM NaCl, 50 mM Tris pH 7.5, and 5 mM TCEP). On-bead cleavage was induced by adding 5 mM CaCl_2_ and incubating the resin at 37 °C for 4 h. After cleavage, the flow-through containing G-MT3 (with one additional Gly residue) was collected and concentrated.

### Acidification and metalation of purified proteins

All the purified MTs were then acidified to pH ~ 2.2 with 7% HCl and concentrated using Amicon Ultra-4 Centrifugal Filter Units with a membrane cut-off of 3 kDa (Merck Millipore, USA) [53]. They were subsequently purified on a size exclusion chromatography SEC-70 gel filtration column (Bio-Rad) equilibrated with 10 mM HCl. The concentration of thiols was determined spectrophotometrically (Jasco V-650 or V-630) using Ellman’s reagent—5,5ʹ-dithiobis-2-nitrobenzoate (DTNB). Ellman’s method involves modification of free thiols in proteins by DTNB and subsequent release of TNB^−^, which is detected spectrophotometrically at 412 nm (ε_412_ = 14,150 M^−1^ × cm^−1^) [54]. DTNB reagent was prepared fresh before use in the concentration of 1 mM in 50 mM HEPES, pH 7.4. Purified thioneins (metal-free or apo-MTs) were mixed with 10 molar excess of ZnSO_4_ and 1 mM TCEP under a nitrogen blanket. TCEP was used here instead of DTT as a disulfide reducing agent of very weakly bound metal ion properties [55]. The pH of the solution was then adjusted to 8.6 with a 1 M Tris solution. Holo-MTs were then concentrated using 3 kDa Amicon Ultra-4 Centrifugal Filter Units (Merck Millipore, USA). Subsequently, they were purified on a SEC-70 gel filtration column (Bio-Rad) equilibrated with 20 mM Tris–HCl, pH 8.6. The total amount of Zn^2+^ bound to holo-MTs was determined using 4-(2-pyridylazo) resorcinol (PAR) assay. When present in excess, PAR binds Zn^2+^ to form Zn(PAR)_2_, which is detected spectrophotometrically at 492 nm (ε_492_ = 71,500 M^−1^ × cm^−1^) [56]. A stock solution of 20 mM PAR was prepared in DMSO for long storage at room temperature. The stock was diluted before use to 100 µM concentration in 50 mM HEPES (pH 7.4) and 1 mM DTNB solution. DTNB was added to modify free thiols and prevent Zn^2+^ rebinding. Concentrations of thiols in holo-MTs were determined spectrophotometrically using 1 mM EDTA in DTNB solution [53]. EDTA is a strong metal chelator that releases metal ions bound to MTs and enables modification of free thiols by DTNB.

### ICP-AES measurements

ICP-AES measurement was done in the Mass Spectrometry Laboratory at the Life Sciences University in Wrocław (Poland). Samples were analyzed by ICP (ICP-AES iCAP 7400, Thermo Scientific). Prior to analysis protein samples were diluted with 0.5 M ultra-pure nitric(V) acid. The final concentration of used MTs was within 10–20 μM. Control samples with 20 mM Tris–HCl, pH 8.6, were also prepared. Calculation of Zn^2+^ molar concentration accounted for the increased density of the solvent.

### Metal binding properties of purified proteins

The titration of purified apo-forms of MT3-WT, GS-MT3, and G-MT3 with Zn^2+^ and Cd^2+^ was monitored spectrophotometrically at 25 °C in the 200–300 nm UV range. 1 µM apo-MT was titrated with 500 μM ZnSO_4_ or CdSO_4_ in chelexed 50 mM borate buffer (100 mM NaClO_4_, pH 7.4) and 100 µM TCEP under anaerobic conditions. The buffer was blanked in a 1 cm quartz cuvette followed by mixing with an appropriate concentration of apo-MT3 solution prepared in 10 mM HCl. The resultant solution was then titrated with subsequent 0.5–1 molar equivalents of 500 μM ZnSO_4_ or CdSO_4_. One accumulation was recorded using a 2 nm bandwidth, a 200 nm per min scanning speed, and a 1.0 nm data pitch. Spectra were averaged from the following two accumulations.

## Results

### Rationale

**Fig. 1:**
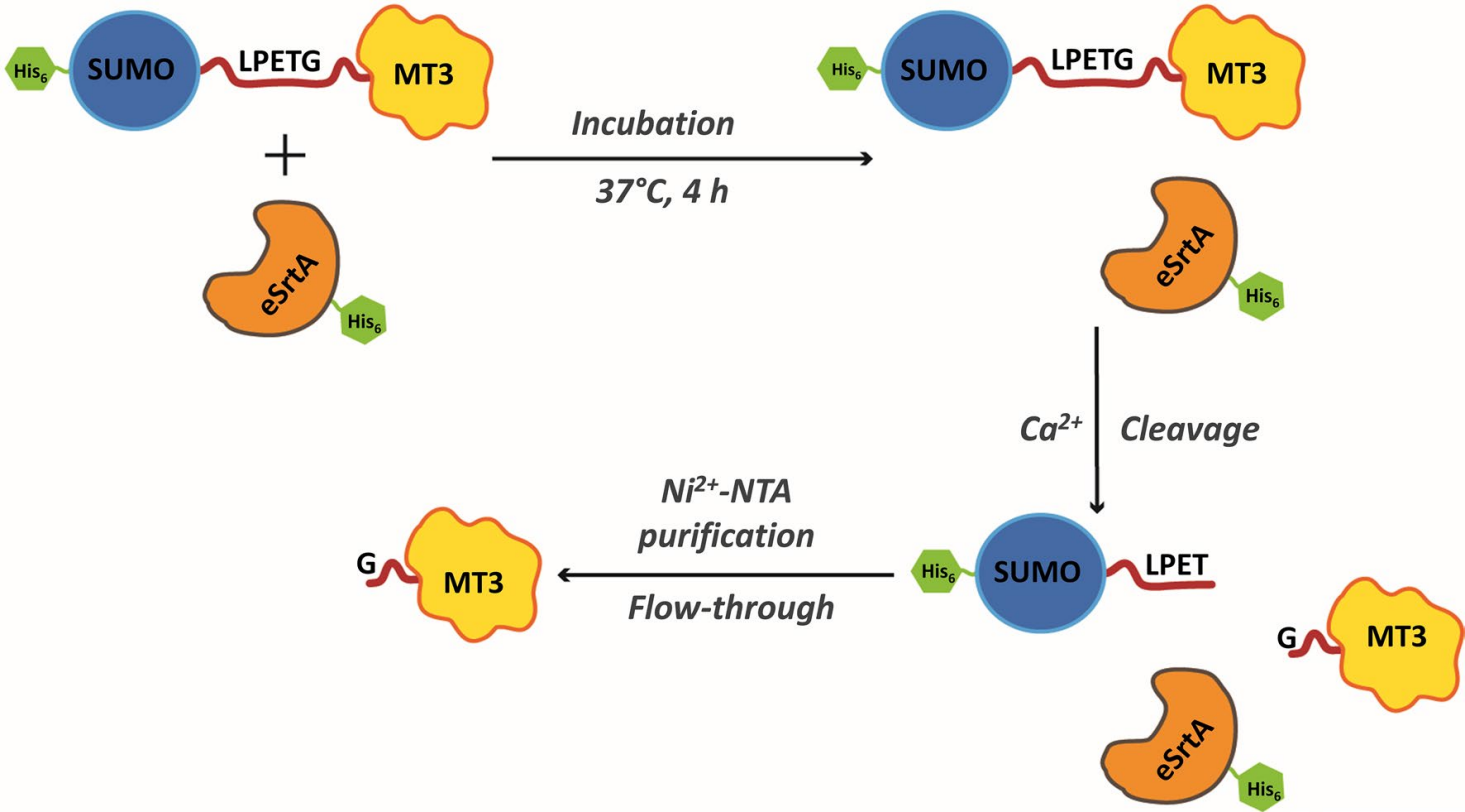
Schematic representation of sortase-mediated purification of SUMOylated human MT3. Purified SUMO-LPETG-MT3 fusion protein was incubated with eSrtA for 4 h at 37 °C in the presence of 5 mM CaCl_2_ for sortase-mediated cleavage of MT3 from the fusion protein. The cleaved G-MT3 was then purified from the reaction mixture using Ni^2+^-IMAC purification

A schematic representation of the sortase-mediated purification of SUMOylated human MT3 is shown in Fig. 1. In the first step, the fusion proteins His_6_-SUMO-LPETG-MT3 and -eSrtA-His_6_ were expressed and purified. Evolved sortase A (eSrtA) is a pentamutant version of *S. aureus* sortase A (SaSrtA) with improved kinetics and activity. It recognizes LPXTG-containing proteins/peptides as its substrate and cleaves at the scissile Thr–Gly peptide bond. In the second step, the purified fusion protein His_6_-SUMO-LPETG-MT3 was incubated with eSrtA-His_6_ for 4 h at 37 °C for sortase-mediated cleavage. After cleavage, the reaction mixture was subjected to Ni^2+^-NTA purification, and the human MT3 having an extra Gly residue at its N-terminal was released while the N-terminal portion of the fusion protein as well as eSrtA remained bound to the Ni^2+^-NTA beads.

**Fig. 2:**
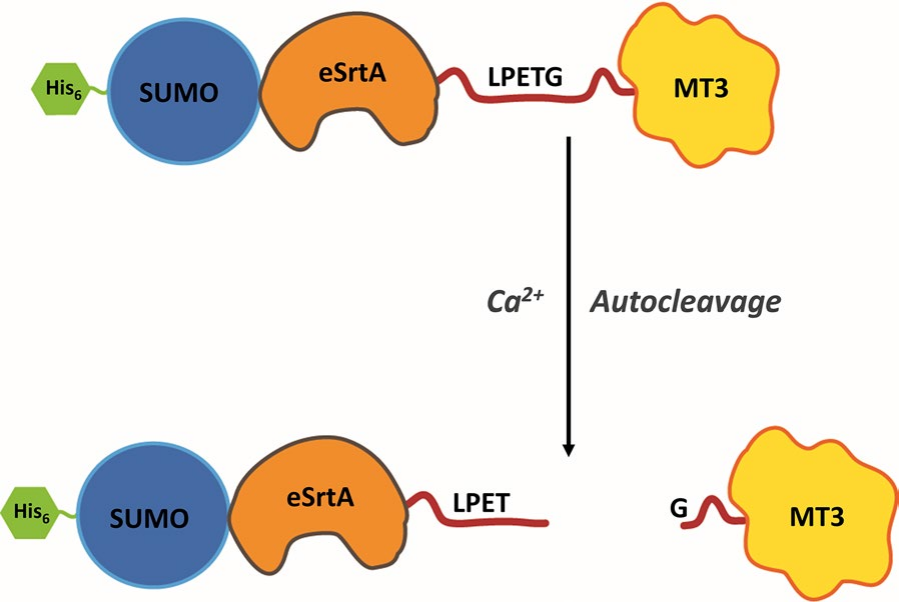
Schematic representation of Ca^2+^-inducible on-bead autocleavage purification of G-MT3 from SUMOylated human MT3. The fusion protein His6-SUMO-eSrtA-LPETG-MT3 was immobilized/bound on Ni^2+^-NTA beads. On-bead sortase-mediated autocleavage was induced by incubating the beads in 5 mM CaCl_2_ for 4 h at 37 °C. Purified G-MT3 was collected in the flow-through

To further improve this purification strategy or to make it a one-step self-cleavable on-bead purification strategy, we designed another fusion protein having eSrtA between SUMO and LPETG. This fusion protein, His_6_-SUMO-eSrtA-LPETG-MT3, was expressed in *E. coli* and purified by Ni^2+^-NTA IMAC. On-column eSrtA-mediated cleavage of the immobilized fusion protein was then achieved by incubating it with Ca^2+^ for 4 h at 37 °C. The target protein with an extra N-terminal glycine residue was released from the fusion while the N-terminal portion remained bound to the Ni^2+^-NTA beads. A schematic representation of this Ca^2+^-induced on-bead autocleavage purification of SUMOylated human MT3 is shown in Fig. 2.

### Plasmid construction

**Fig. 3:**
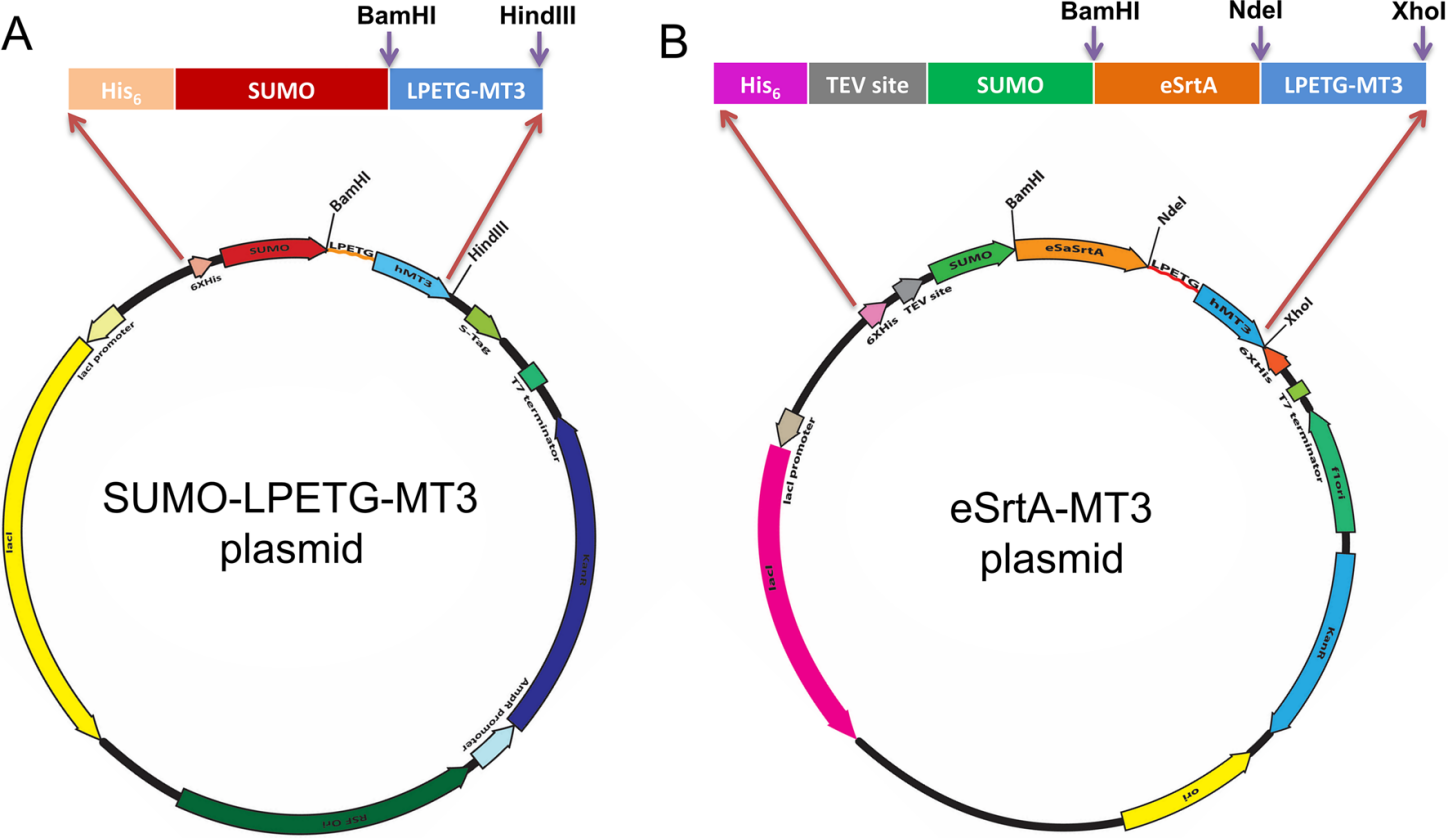
Plasmid map of the constructed expression vectors encoding fusion proteins SUMO-LPETG-MT3 and sSrtA-MT3

The plasmid pBHRSF184 (Addgene plasmid #89482), which expresses the N-terminally His-tagged SUMO-OaAEP1 [33] fusion protein, was used as a template for the generation of SUMO-MT3 and SUMO-LPETG-MT3 plasmids. The gene coding for the MT3 protein was amplified using MT3-BamHI-Fwd and MT3-HindIII-Rev primers from the MT3-pTYB21 plasmid. To insert the sortase recognition motif LPETG at the N-terminus of MT3 protein, a coding region for LPETG was inserted in the sequence of the MT3-BamHI-Fwd primer. The resultant PCR-amplified genes coding for MT3 and LPETG-MT3 were inserted into the pBHRSF184 vector between BamHI and HindIII sites, resulting in SUMO-MT3 (Addgene plasmid #200303) and SUMO-LPETG-MT3 plasmids (Addgene #200304) respectively. These recombinant vectors have an upstream T7 promoter followed by the fusion protein with an N-terminal His-tagged SUMO (Fig. 3A).

Similarly, the plasmid pETM11-SUMO-SNCA-GFP (Addgene plasmid # 107292), which expresses N-terminally His-tagged SUMO-α-synuclein-eGFP fusion protein [50], was used as a template for generation of the eSrtA-MT3 plasmid (Fig. 3B) in a two-step process (see Materials and methods). This recombinant eSrtA-MT3 plasmid (Addgene #200305) has an upstream T7 promoter followed by the fusion protein consisting of an N-terminal His-tag, TEV cleavage site, SUMO, pentamutant sortase A (eSrtA), the sortase recognition motif LPETG and MT3 with a stop codon (Fig. 3B). Pentamutant evolved sortase A (eSrtA) was added for the one-step on-bead self-cleavage and purification of the MT3 protein.

### Expression and purification of proteins

**Fig. 4:**
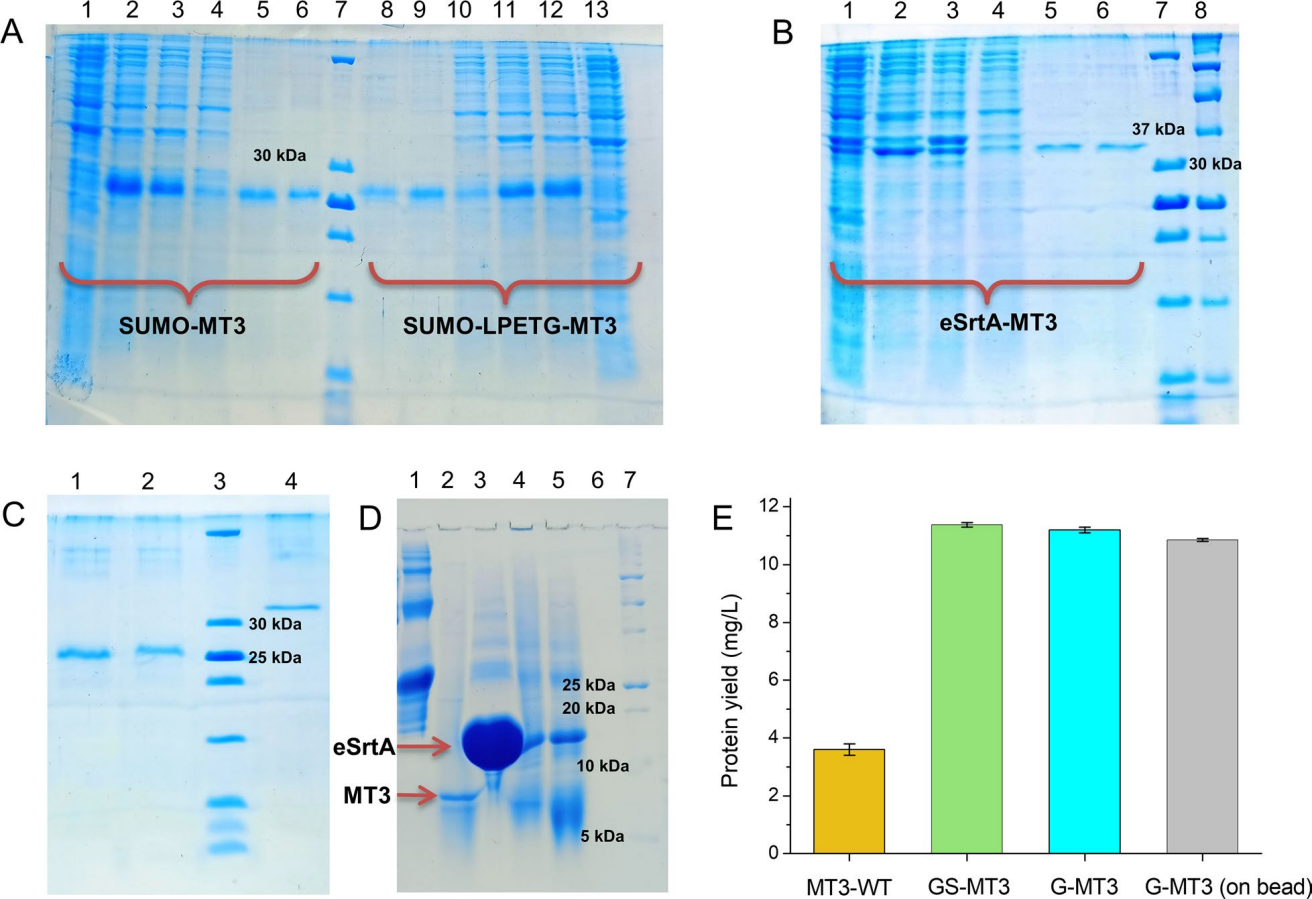
Purification of fusion proteins. **A** SDS-PAGE purification profile of SUMO-MT3 and SUMO-LPETG-MT3. Lane 1 = lysate, Lane 2 = supernatant, Lane 3 = flow-through, Lane 4 = wash, Lane 5 = first elution, Lane 6 = second elution, Lane 7 = protein ladder, Lane 8 = second elution, Lane 9 = first elution, Lane 10 = wash, Lane 11 = flow-through, Lane 12 = supernatant, Lane 13 = lysate. **B** SDS-PAGE purification profile of eSrtA-MT3. Lane 1 = lysate, Lane 2 = supernatant, Lane 3 = flow-through, Lane 4 = wash, Lane 5 = first elution, Lane 6 = second elution, Lane 7 = protein ladder, Lane 8 = protein ladder Bio-Rad. **C** SDS-PAGE profile of purified fusion proteins. Lane 1 = SUMO-MT3, Lane 2 = SUMO-LPETG-MT3, Lane 3 = protein ladder, Lane 4 = eSrtA-MT3. **D** SDS-PAGE profile of eSrtA mediated cleavage of SUMO-LPETG-MT3. Lane 1 = fusion protein SUMO-LPETG-MT3, Lane 2 = eSrtA cleaved MT3, Lane 3 = SUMO eluted from beads after cleavage, Lane 4 = proteins from beads, Lane 5 = fusion protein in eSrtA buffer, Lane 6 = empty, and lane 7 = protein ladder Bio-Rad. **E** Comparison of total protein yield (mg/L) obtained using various purification systems. MT3-WT stands for protein obtained from the IMPACT system

All the proteins were expressed and purified from their respective plasmids as described in Materials and Methods. The amino acid sequence of the fusion constructs and purified MTs can be found in the supplementary information (Additional file 1: Figs. S1-S4). Samples from each step of the purification were collected and analyzed on SDS-PAGE (Fig. 4A–D). The SDS-PAGE purification profile of the fusion protein eSrtA-MT3 (Fig. 4B and C) showed a single band at 35 kDa for the fusion protein. The masses of the fusion proteins were in accordance with their molecular weights (Additional file 1: Table S1, Fig. 4A–C). Similarly, the purification profile (Fig. 4A and C) of SUMO-MT3 and SUMO-LPETG-MT3 fusion proteins showed a single band at 25 kDa that migrated in accordance with the molecular weight, indicating homogeneity of the protein preparation. GS-MT3 was obtained from SUMO-MT3 fusion protein by Ulp1 (37 µM)-mediated cleavage and second Ni^2+^-NTA purification. Similarly, G-MT3 was obtained from the fusion protein SUMO-LPETG-MT3 by eSrtA (100 µM)-mediated cleavage as described above. The sortase-mediated cleavage and Ni^2+^-NTA purification of G-MT3 were analyzed on SDS-PAGE (Fig. 4D). The SDS-PAGE showed the cleavage of G-MT3 from SUMO-LPETG-MT3 as it migrated as a single band of 7 kDa. The SUMO tag, which remained bound to the Ni^2+^-NTA, was eluted with 350 mM imidazole and was also analyzed (Fig. 4D). To purify G-MT3 from the eSrtA-MT3 construct, 5 mM CaCl_2_ was added to the Ni^2+^-NTA resin bound fusion protein His_6_-SUMO-eSrtA-LPETG-MT3 to activate the sortase-mediated auto-cleavage. It was observed that on the bead, autocleavage was very slow. The addition of 10 µM eSrtA increased the rate of sortase-mediated autocleavage and resulted in higher protein yield (Additional file 1: Fig. S5). Inducible on-bead sortase-mediated cleavage of G-MT3 from eSrtA-MT3 in the presence/absence of 5 mM CaCl_2_ was also analyzed on SDS-PAGE (Additional file 1: Fig. S5). The SDS-PAGE showed the successful on-bead cleavage of G-MT3, a small amount of self-cleavage in the absence of CaCl_2_ was also observed, as per expectations. After cleavage, G-MT3 protein was collected in the flow-through and was further purified using size exclusion chromatography (SEC) with a SEC-70 gel filtration column (Bio-Rad) equilibrated with 10 mM HCl (Additional file 1: Fig. S6). MT3-WT, GS-MT3, and G-MT3 obtained from SUMO-LPETG-MT3 were also purified using size exclusion chromatography (SEC) in 10 mM HCl to avoid protein oxidation during separation (Additional file 1: Fig. S6). The mass of purified apo-MTs (MT3-WT, GS-MT3, and G-MT3) and eSrtA was confirmed by electrospray ionization mass spectrometry (ESI–MS) (Additional file 1: Fig. S7). Freshly prepared Ellman’s reagent (5,5ʹ-dithiobis-2-nitrobenzoate – DTNB) was used to determine the concentration of spectrophotometric thiols in the SEC purified proteins. Purified protein overall yield was calculated based on these concentrations and the volume. The overall yield for the MT3-WT purified using the IMPACT system was 3.4 mg/L, while the yield for GS-MT3 purified from SUMO-MT3 fusion protein by Ulp1-mediated cleavage was 11.5 mg/L (Fig. 4E). The overall yield of G-MT3 purified from SUMO-LPETG-MT3 was 11.0 mg/L. The yield of G-MT3 obtained from on-bead sortase-mediated cleavage of SUMO-eSrtA-LPETG-MT3 was 6.0 mg/L and 10.8 mg/L (with 10 µM eSrtA), respectively (Fig. 4E and Additional file 1: Fig. S5).

To prepare Zn^2+^-loaded forms (holo-forms), the fractions containing freshly prepared apo-proteins were collected and metalated by ZnSO_4_ excess addition (up to 115% of expected loading state) in the presence of 1 mM TCEP under a nitrogen blanket. TCEP was used here as a disulfide reducing agent with very low metal binding abilities, compared to e.g. DTT [55, 57]. For metalation, the pH was increased to 8.6 using 1 M Tris base to increase complex stability [29]. Zn^2+^-loaded samples were concentrated using a 3 kDa Amicon centrifugal filter and holo-proteins were purified on a SEC column equilibrated with 20 mM Tris, pH 8.6. The SEC fractions having holo-MTs were concentrated, and aliquots were stored at − 80 °C until further use.

### Determination of Zn^2+^-to-protein stoichiometry

The stoichiometry of Zn^2+^ complexes obtained after reconstitution and purification of holo-forms was determined using two different approaches. In the first one, two spectroscopic assays were applied to determine concentrations of total thiol(ates) and Zn^2+^. Thiol(ates) were determined by DTNB in the presence of EDTA and Zn^2+^ by PAR (4-(2-pyridylazo)resorcinol) in the presence of DTNB. The addition of EDTA in the first and DTNB in the second increase the reaction rates. Thiol(ate) concentration was then transformed into protein concentration. It was possible to assume that all Cys residues were reduced due to fresh reduction and preparation. The determined molar ratios between Zn^2+^ and protein concentrations are presented in Table 1. In the second approach, protein samples were wet mineralized in nitric acid and analyzed for zinc content by inductively coupled plasma atomic emission spectroscopy (ICP-AES). Obtained Zn^2+^ molar concentrations were divided by molar protein concentrations to obtain ratios analogous to spectroscopic assays (Table 1). Results from both approaches are highly convergent despite methodological differences.

**Table 1:** ICP and spectroscopic analysis of Zn^2+^-to-protein molar ratios in reconstituted MT2-WT, MT3-WT, GS-MT3, and G-MT3 obtained in this study (MT2 was used as control)

Protein	ICP analysis Zn^2+^/protein	Spectroscopic analysis Zn^2+^/protein	Average Zn^2+^/protein
MT2-WT	7.1 ± 0.1	7.0 ± 0.2	7.0 ± 0.3
MT3-WT	7.0 ± 0.1	6.9 ± 0.1	7.0 ± 0.2
GS-MT3	7.3 ± 0.1	6.9 ± 0.2	7.1 ± 0.3
G-MT3	7.1 ± 0.3	6.8 ± 0.2	7.0 ± 0.5

### UV–vis spectroscopy

**Fig. 5:**
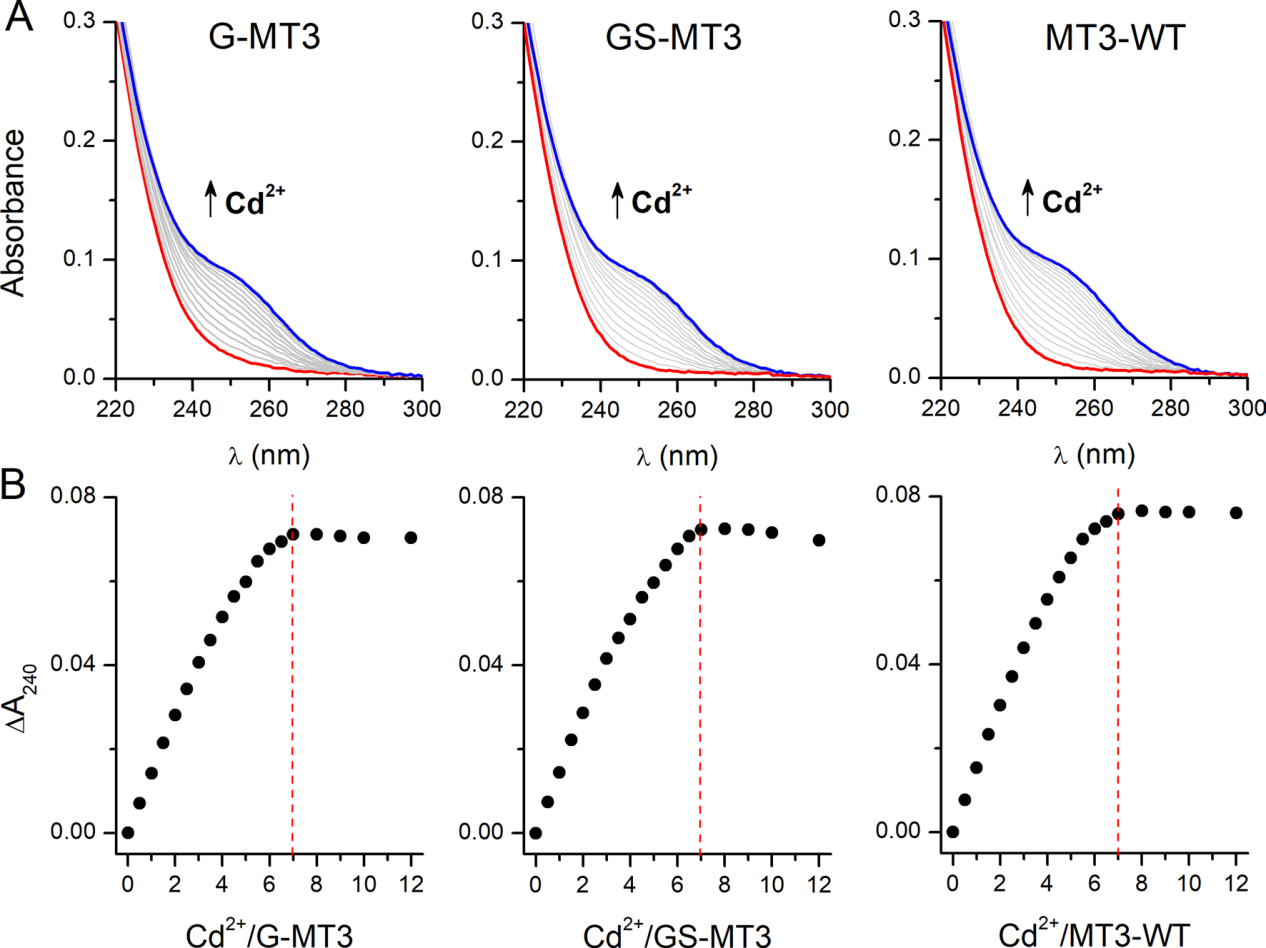
UV–vis monitored Cd^2+^ titration of (**A**) 1 µM metal-free G-MT3, GS-MT3, and MT3-WT in 50 mM borate pH 7.4, 100 mM NaClO_4_, 100 µM TCEP. Panel (**B**) indicates saturation isotherms recorded at 240 nm for CdSO_4_ titrations. Red vertical lines indicate metal-to-protein ratios where absorbance reaches saturation with Cd^2+^

UV–vis spectroscopy is the most common technique used to determine metal binding properties and metalation status of MTs. MT spectra demonstrate ligand-to-metal charge transfer (LMCT) bands in UV regions between 200 and 400 nm. Usually in proteins, this region is masked by the presence of aromatic groups and disulfide bonds but MTs lack both of them, making it easy to observe the LMCT absorption bands. The maxima of LMCT bands for Zn^2+^ are observed around 220 nm while those for Cd^2+^ are around 240 nm. To monitor the LMCT transition bands during metalation the UV–vis spectra in the wavelength range of 220–300 nm were recorded for apo-forms of MT3-WT, GS-MT3, and G-MT3. Firstly, the absorbance was blanked using 50 mM borate buffer (100 mM NaClO_4_, pH 7.4) with 100 µM TCEP followed by the addition of 1 µM apo-MT to record the spectrum for apo-protein, which has the lowest intensity. An increase in absorbance was observed with the addition of successive molar equivalents of ZnSO_4_ or CdSO_4_ until the apo-MTs were saturated with these divalent metal ions. Addition of ZnSO_4_ or CdSO_4_ above saturation points does not cause any absorbance increase (Fig. 5A, Additional file 1: Fig. S8). The absorbance difference was obtained by subtracting the absorbance of apo-MT from the absorbance at different molar equivalents of the metal ion. A scatter graph showing the dependence of absorbance on metal to apo-MT molar ratio was plotted for Cd^2+^ at 240 nm (Fig. 5B). It shows that protein saturation with Cd^2+^ occurs at 7 Cd^2+^ mol. eq., as expected. All obtained proteins here show the same metal binding properties, indicating that they are functional in terms of metal binding properties.

## Discussion

The complex array of intracellular processes involved in protein expression includes transcription, protein folding, post-translation processing, and secretion. Challenges in any of these steps could result in low protein expression, while the inherent properties of the molecule itself may limit its production via mechanisms such as cytotoxicity or inherent instability. MTs are cysteine-rich and have a modest molecular mass but still, it is challenging to directly express them by genetic engineering techniques. Expression levels are quite low whether yeast or *E. coli* is utilized for this purpose [20, 28, 58]. Although MT protein solubility and expression levels have been dramatically enhanced thanks to fusion expression techniques, monomeric MT yield was noticeably lower than that of polymer MTs [20, 28]. As a molecular chaperone, SUMO has recently been discovered to boost the solubility and expression levels of heterogeneous proteins [38, 39, 45]. By offering a very hydrophobic core that serves as a nucleation site for target protein folding and encourages interactions between proteins and proper protein folding, it has also been discovered that the SUMO protein increases the solubility of fusion proteins [39, 59, 60]. The present study presents for the first time a combinatorial approach using SUMO and/or sortase as a fusion tag for high-level expression of human MT in *E. coli* and its purification by three different strategies. In the first strategy, SUMOylated human MT3 was expressed and purified using Ni^2+^-NTA. MT3 containing two additional residues (GS-MT3) at its N-terminus was obtained from this purified SUMOylated human MT3 by Ulp1-mediated cleavage and second Ni^2+^-NTA purification. The yield of apo-GS-MT3 obtained was found to be 11.5 mg/L, which was 3.5-fold higher than the yield of apo-MT3 purified using the IMPACT system (3.4 mg/L). This increase in total protein yield of apo-GS-MT3 is fully supported by previous studies where SUMO has been used as a fusion tag for high-level expression of toxic and difficult-to-express proteins. SUMO itself, a rapidly folding, stable protein, helps serve as an initiation site for the folding of the partner protein. But one of the drawbacks of this purification strategy, in addition to the use of a SUMO protease (Ulp1), is that the final purification product (apo-GS-MT3) contains two additional residues. To overcome this limitation of Ulp1-mediated purification of human MT3 we shifted our focus to a bacterial cysteine transpeptidase known as sortase. Sortases are responsible for the covalent anchoring of essential surface proteins on the bacterial cell wall or peptidoglycan [61–63]. *S. aureus* sortase A (SaSrtA) has emerged as a protein engineer’s versatile tool for protein labeling, circularization, modification, semi-synthesis, and protein–protein fusions [51, 64, 65]. SaSrtA recognizes an LPXTG pentapeptide motif by an elongated hydrophobic groove at its active site, cleaves the T − G peptide bond through a nucleophilic attack from the side chain of its catalytic Cys184, and forms an acyl-enzyme intermediate, which is then resolved by another nucleophilic attack of an incoming aminoglycine [66–68]. eSrtA is a pentamutant variant of SaSrtA (5 M SrtA) and has reportedly 140-fold higher activity. In the second strategy, we incorporated a sortase recognition motif (LPETG) at the N-terminus of MT3, and the resultant SUMOylated LPETG-MT3 was expressed in *E. coli* and purified using Ni^2+^-NTA resin, which was not affected by fusion protein (MTs bind divalent metal ions). After purification, this fusion protein was treated with eSrtA for sortase-mediated cleavage of the Thr-Gly peptide bond of the LPETG motif. MT3 containing one additional Gly residue (G-MT3) at its N-terminus was obtained after the second Ni^2+^-NTA purification. The yield of apo-G-MT3 obtained was found to be 11 mg/L. This strategy further reduced the number of additional residues from two to one, but it was still a two-step purification process and required the addition of purified eSrtA from outside for the sortase-mediated purification of G-MT3. So, to further improve this strategy and to make it a one-step on-bead self-cleavable purification process, we added the eSrtA enzyme as a fusion partner in our fusion protein between SUMO and LPETG-MT3. The presence of eSrtA in the fusion protein not only provides on-column inducible self-cleavage but has also been reported to promote the fusion expression of poorly expressed proteins [46]. The resultant fusion protein His_6_-SUMO-eSrtA-LPETG-MT3 was expressed in *E. coli* and purified using Ni^2+^-NTA. After the binding of the fusion protein to Ni^2+^-NTA resin, the resin was washed 2–3 times and auto-cleavage was induced by adding 5 mM CaCl_2_. The addition of 10 µM eSrtA along with 5 mM CaCl_2_ increased the rate of sortase-mediated autocleavage and resulted in higher protein yield. The addition of CaCl_2_ does not affect the binding efficiency of Ni^2+^-NTA resin [46]. The MT3 containing one additional Gly residue (G-MT3) at its N-terminus was obtained in the flow-through after cleavage while the rest of the fusion protein remained bound to the resin. The yield of apo-G-MT3 obtained was 10.8 mg/L. This on-bead self-cleavage sortase-based protein purification strategy was first demonstrated for emerald GFP (emGFP). In this report, it was also observed that the fusion of sortase at the N-terminal of emGFP enhanced its expression level compared to emGFP without fusion [46]. So, to check/show the effect of SUMO on the expression level of His_6_-SUMO-eSrtA-LPETG-MT3 (eSrtA-MT3 fusion protein), another construct His_6_-eSrtA-LPETG-MT3 (eSrtA-MT3 without SUMO) was generated and used as a control (Additional file 1: Figure S9). This construct's expression level and solubility (eSrtA-MT3 without SUMO) were lower than that of eSrtA-MT3. A large fraction of the expressed control protein was found in the pellet fraction. Loss of fusion protein during the washing step (with 50 mM imidazole) was also seen (Additional file 1: Figure S9). The presence of both SUMO and sortase in the fusion construct significantly increased its expression in *E. coli*, while the presence of sortase and its recognition motif LPETG provided the opportunity for inducible on-bead self-cleavage for protein purification. This inducible on-bead self-cleavage can further be improved by incorporating small linkers between the eSrtA and the LPETG recognition motif, which will increase the flexibility of this region and will make the recognition motif easily accessible to the sortase catalytic site. The three strategies reported in the current article for the purification of human MT3 were also compared with the MT expression/purification strategies previously reported by different groups and the pros and cons of these strategies were analyzed. The comparison along with the respective references can be found in Table 2. The Ca^2+^-induced on-bead autocleavage purification is a very simple, cost-effective, fast, and efficient strategy that resulted in a very high expression and purification yield of MT3. The MT3 yield obtained using this strategy is almost threefold higher than the yield of MT3 obtained using the IMPACT system. Also, the MT3 obtained using this strategy contains only one additional glycine at its N-terminus as compared to the 6–8 residues left after GST or S-tag-based purification. Another advantage of this strategy is that it is a one-step purification process in which on-bead autocleavage is induced by Ca^2+^ and there is no need for any external protease. The smaller number of steps during purification avoids the unnecessary loss of the protein and results in a higher yield.

**Table 2:** Comparison of different MT purification strategies along with their advantages and disadvantages. MT refers to mammalian metallothionein if not specified otherwise

Purification tag or system	Yield (isoform)	Advantages	Disadvantages	References
IMPACT	6 mg/L (MT2) 3.8 mg/L (MT3)	No additional amino acid residues left	Time consuming, expensive	[28, 48] This study
GST	5.12 mg/L (MT2)		Six amino acid residues are left after cleavage of GST tag	[20]
Β-galactosidase	(MT2)	Used as biosorbent for waste-water treatment	No recombinant protein purified	[69]
GST-SUMO	(MT1A)	Fusion protein effectively protects against oxidative damage	Apo-MT not purified from fusion protein	[45]
Ubiquitin	yeast MT	Ub fusion stabilizes the MT	Apo-MT not purified from fusion protein	[58]
S-tag	(MT1A)		6–8 amino acid residues are left after S-tag removal	[26, 27]
Tagless	(MT2)	No additional residues left	Requires several SEC and ion-exchange steps	[30–32]
SUMO-MT3	11.5 mg/L (MT3)	Cost-effective high yield purification, simple, fast and efficient	Two amino acid residues left after SUMO tag removal	This study
SUMO-LPETG-MT3	11 mg/L (MT3)	Cost-effective high yield purification, simple, fast and efficient	One Gly residue left after sortase cleavage	This study
SUMO-eSrtA-LPETG-MT3	10.8 mg/L (MT3)	On-bead inducible auto-cleavage purification, cost-effective, fast and efficient	One Gly residue left after sortase cleavage	This study

Ni^2+^-NTA IMAC purification is at present the most effective, simple, and inexpensive protein purification technique available and is the first choice of any researcher. However, it is rarely used for the purification of MTs. Since MTs can bind divalent M^2+^, the possibility of Ni^2+^ interference during purification as well as further biochemical characterization of MTs remains an issue. In our current strategy, we have used two fusion proteins, SUMO and eSrtA, between N-terminal His_6_-tag and C-terminal MT, and hence the chance of any influence of Ni^2+^-NTA on MTs is very low. Moreover, we also compared the metal binding properties of the MTs purified using the current strategies as well as by the IMPACT system, and they were almost identical. All the proteins bind seven Cd^2+^ ions, as shown by the UV–Vis metal titration, and seven Zn^2+^ ions, as confirmed by ICP and DTNB/PAR assays. Hence, these results also suggest that the current Ni^2+^-NTA-based purification strategy does not influence the metal content of the recombinant MTs. However, applying the approaches presented here for other MTs requires initial testing of how particular proteins (may differ in metal affinity) potentially interact with Ni^2+^-NTA to avoid Ni^2+^ removal by MT and lower overall production yield.

## Future outlooks

SUMO-sortase fusion construct-based strategy, described in this study, is a simple, robust, and inexpensive one-step purification approach for metallothioneins via IMAC. This strategy's inducible on-bead self-cleavage efficiency can be further improved by increasing the flexibility between eSrtA and its recognition motif LPETG. This strategy can be further extended to the purification of other difficult-to-express and toxic mammalian or viral proteins.

## Supplementary Information

**Additional file 1: Figure S1**. Sequences of fusion construct and purified WT-MT3. The WT-MT3 was expressed as a fusion protein with Chitin Binding Domain and Intein. The WT-MT3 (without any additional residues) was purified by IMPACT purification. **Figure S2**. Sequences of SUMO-MT3 fusion construct and purified GS-MT3. The GS-MT3 was expressed as a fusion protein with His_6_-SUMO. The fusion protein was purified by Ni^2+^-NTA and was then treated with Ulp1. The GS-MT3 (with extra Gly-Ser residues) was collected in flow through of the second Ni^2+^-NTA purification. **Figure S3**. Sequences of SUMO-LPETG-MT3 fusion construct and purified G-MT3. The LPETG-MT3 was expressed as a fusion protein with His_6_-SUMO. The fusion protein was purified by Ni^2+^-NTA and was then treated with eSrtA. The G-MT3 (with extra Gly residue) was collected in flow through of the second Ni^2+^-NTA purification. **Figure S4**. Sequences of eSrtA-MT3 fusion construct and purified G-MT3. This protein was expressed as a fusion protein having His_6_-SUMO-eSrtA-LPETG-MT3. The fusion protein was loaded on Ni^2+^-NTA resin and on-bead sortase-mediated auto-cleavage was induced by adding 5 mM CaCl_2_. The G-MT3 (with extra Gly residue) was collected in flow-through. **Figure S5**. Inducible on-bead sortase-mediated cleavage of eSrtA-MT3. A) SDS-PAGE profile of on-bead sortase-mediated cleavage of eSrtA-MT3. Lane 1 = protein ladder, Lane 2 = fusion protein after binding to the Ni^2+^-NTA beads, Lane 3 = cleavage of eSrtA-MT3 in the absence of Ca^2+^ ion, Lane 4 = cleavage of eSrtA-MT3 in the presence of Ca^2+^ ion, Lane 5 = purified G-MT3, Lane 6 = uncleaved eSrtA-MT3 left on the Ni^2+^-NTA beads, and lane 7 = protein ladder. B) Comparison of total protein yield (mg/L) obtained during Ca^2+^ induced on-bead cleavage of the eSrtA-MT3 in the presence and absence of external 10 µM eSrtA. **Figure S56**. SEC purification profile of apo-forms of WT-MT3, GS-MT3 (with additional Gly-Ser) and G-MT3 (with additional Gly) on a Superdex™ 75 Increase 10/300 GL column using 10 mM HCl, and absorbance was recorded at 220 and 280 nm. The retention volume for all apo-MTs was 19 mL. **Figure S7**. Deconvoluted ESI-MS spectra of WT-MT3, GS-MT3, G-MT3, and eSrtA obtained in this study. **Figure S8**. UV-vis monitored Zn(II) titrations to 1 µM metal-free G-MT3, GS-MT3, and MT3-WT in 50 mM borate pH 7.4, 100 mM NaClO_4_, 100 µM TCEP. **Figure S9**. SDS-PAGE purification profile of His_6_-eSrtA-LPETG-MT3 (eSrtA-MT3 without SUMO). Lane 1 = pellet, Lane 2 = lysate, Lane 3 = supernatant, Lane 4 = flowthrough, Lane 5 = wash, Lane 6 = eluted G-MT3, and lane 7 = protein ladder. A large fraction of the expressed protein was found in the pellet fraction. Loss of fusion protein during the washing step (with 50 mM imidazole) was also seen. Lane 8: A reference protein ladder (the same ladder as in lane 7). **Table S1**. Molecular masses and extinction coefficients of fusion constructs and purified proteins. Experimental masses were obtained from ESI-MS measurements. **Table S2**. List of all the primers used in this study.

## Data Availability

All data generated and analyzed in this study are included in this article and Additional files. The plasmids generated in this study have been submitted to the Addgene (Id #200303, 200304, and 200305).
