# Supplementary material for: Calcium-assisted sortase A cleavage of SUMOylated metallothionein constructs leads to high-yield production of human MT3
Source: Microb Cell Fact. 2023 Jul 11;22:125. doi: 10.1186/s12934-023-02134-x (PMC10334657; doi:10.1186/s12934-023-02134-x)
Supplement: Supplementary file 1 — Additional file 1: Figure S1. Sequences of fusion construct and purified WT-MT3. The WT-MT3 was expressed as a fusion protein with Chitin Binding Domain and Intein. The WT-MT3 (without any additional residues) was purified by IMPACT purification. Figure S2. Sequences of SUMO-MT3 fusion construct and purified GS-MT3. The GS-MT3 was expressed as a fusion protein with His6-SUMO. The fusion protein was purified by Ni2+-NTA and was then treated with Ulp1. The GS-MT3 (with extra Gly-Ser residues) was collected in flow through of the second Ni2+-NTA purification. Figure S3. Sequences of SUMO-LPETG-MT3 fusion construct and purified G-MT3. The LPETG-MT3 was expressed as a fusion protein with His6-SUMO. The fusion protein was purified by Ni2+-NTA and was then treated with eSrtA. The G-MT3 (with extra Gly residue) was collected in flow through of the second Ni2+-NTA purification. Figure S4. Sequences of eSrtA-MT3 fusion construct and purified G-MT3. This protein was expressed as a fusion protein having His6-SUMO-eSrtA-LPETG-MT3. The fusion protein was loaded on Ni2+-NTA resin and on-bead sortase-mediated auto-cleavage was induced by adding 5 mM CaCl2. The G-MT3 (with extra Gly residue) was collected in flow-through. Figure S5. Inducible on-bead sortase-mediated cleavage of eSrtA-MT3. A) SDS-PAGE profile of on-bead sortase-mediated cleavage of eSrtA-MT3. Lane 1 = protein ladder, Lane 2 = fusion protein after binding to the Ni2+-NTA beads, Lane 3 = cleavage of eSrtA-MT3 in the absence of Ca2+ ion, Lane 4 = cleavage of eSrtA-MT3 in the presence of Ca2+ ion, Lane 5 = purified G-MT3, Lane 6 = uncleaved eSrtA-MT3 left on the Ni2+-NTA beads, and lane 7 = protein ladder. B) Comparison of total protein yield (mg/L) obtained during Ca2+ induced on-bead cleavage of the eSrtA-MT3 in the presence and absence of external 10 µM eSrtA. Figure S56. SEC purification profile of apo-forms of WT-MT3, GS-MT3 (with additional Gly-Ser) and G-MT3 (with additional Gly) on a Superdex™ 75 Increase 10/300 [file 12934_2023_2134_MOESM1_ESM.docx]

*Electronic supplementary information*

**Calcium-assisted sortase A cleavage of SUMO-ylated metallothionein constructs leads to high-yield production of human MT3**

Avinash Kumar Singh and Artur Krężel*

*Department of Chemical Biology, Faculty of Biotechnology University of Wrocław, Joliot-Curie 14a, 50-383 Wrocław, Poland*

*Correspondence to A. Krężel, E-mail: artur.krezel@uwr.edu.pl

**Table of contents**

Figure S1. Sequences of fusion construct and purified WT-MT3⋅⋅⋅⋅⋅⋅⋅⋅⋅⋅⋅⋅⋅⋅⋅⋅⋅⋅⋅⋅⋅⋅⋅⋅⋅⋅⋅⋅⋅⋅⋅⋅⋅⋅⋅⋅⋅⋅⋅⋅⋅⋅⋅⋅⋅⋅⋅⋅⋅⋅⋅⋅⋅⋅⋅⋅⋅⋅⋅ S3

Figure S2. Sequences of SUMO-MT3 fusion construct and purified GS-MT3 ⋅⋅⋅⋅⋅⋅⋅⋅⋅⋅⋅⋅⋅⋅⋅⋅⋅⋅⋅⋅⋅⋅⋅⋅⋅⋅⋅⋅⋅⋅⋅⋅⋅⋅⋅⋅⋅ S4

Figure S3. Sequences of SUMO-LPETG-MT3 fusion construct and purified G-MT3 ⋅⋅⋅⋅⋅⋅⋅⋅⋅⋅⋅⋅⋅⋅⋅⋅⋅⋅⋅⋅⋅⋅⋅⋅⋅⋅ S5

Figure S4. Sequences of eSrtA-MT3 fusion construct and purified G-MT3 ⋅⋅⋅⋅⋅⋅⋅⋅⋅⋅⋅⋅⋅⋅⋅⋅⋅⋅⋅⋅⋅⋅⋅⋅⋅⋅⋅⋅⋅⋅⋅⋅⋅⋅⋅⋅⋅⋅⋅⋅⋅⋅ S6

Figure S5. Inducible on-bead sortase-mediated cleavage of eSrtA-MT3⋅⋅⋅⋅⋅⋅⋅⋅⋅⋅⋅⋅⋅⋅⋅⋅⋅⋅⋅⋅⋅⋅⋅⋅⋅⋅⋅⋅⋅⋅⋅⋅⋅⋅⋅⋅⋅⋅⋅⋅⋅⋅⋅⋅⋅⋅⋅⋅⋅S7

Figure S6. SEC purification profile of thioneins of WT-MT3, GS-MT3 and G-MT3⋅⋅⋅⋅⋅⋅⋅⋅⋅⋅⋅⋅⋅⋅⋅⋅⋅⋅⋅⋅⋅⋅⋅⋅⋅⋅⋅⋅ S8

Figure S7. Mass-spectra of WT-MT3, GS-MT3, G-MT3 and eSrtA ⋅⋅⋅⋅⋅⋅⋅⋅⋅⋅⋅⋅⋅⋅⋅⋅⋅⋅⋅⋅⋅⋅⋅⋅⋅⋅⋅⋅⋅⋅⋅⋅⋅⋅⋅⋅⋅⋅⋅⋅⋅⋅⋅⋅⋅⋅⋅⋅⋅⋅⋅⋅⋅ S9

Figure S8. UV-Vis spectra of Zn(II) titrations to thioneins of WT-MT3, GS-MT3 and G-MT3⋅⋅⋅⋅⋅⋅⋅⋅⋅⋅⋅ S10

Figure S9. SDS-PAGE purification profile of His_6_-eSrtA-LPETG-MT3⋅⋅⋅⋅⋅⋅⋅⋅⋅⋅⋅⋅⋅⋅⋅⋅⋅⋅⋅⋅⋅⋅⋅⋅⋅⋅⋅⋅⋅⋅⋅⋅⋅⋅⋅⋅⋅⋅⋅⋅⋅⋅⋅ S11

Table S1. Experimental and expected molecular masses of fusion constructs and purified proteins ⋅⋅⋅⋅⋅S12

Table S2. List of all the primers used in this study⋅⋅⋅⋅⋅⋅⋅⋅⋅⋅⋅⋅⋅⋅⋅⋅⋅⋅⋅⋅⋅⋅⋅⋅⋅⋅⋅⋅⋅⋅⋅⋅⋅⋅⋅⋅⋅⋅⋅⋅⋅⋅⋅⋅⋅⋅⋅⋅⋅⋅⋅⋅⋅⋅⋅⋅⋅⋅⋅⋅⋅⋅⋅⋅⋅⋅⋅⋅⋅⋅⋅⋅⋅⋅⋅⋅⋅⋅⋅⋅⋅ S13

The sequence of CBD-Intein-MT3 fusion protein:

MKIEEGKLVIGSLEGCFAKGTNVLMADGSIECIENIEVGNKVMGKDGRPREVIKLPRGRETMYSVVQKSQHRAHKSDSSREVPELLKFTCNATHELVVRTPRSVRRLSRTIKGVEYFEVITFEMGQKKAPDGRIVELVKEVSKSYPISEGPERANELVESYRKASNKAYFEWTIEARDLSLLGSHVRKATYQTYAPILYENDHFFDYMQKSKFHLTIEGPKVLAYLLGLWIGDGLSDRATFSVDSRDTSLMERVTEYAEKLNLCAEYKDRKEPQVAKTVNLYSKVVRGASTNPGVSAWQVNTAYTAGQLVTYNGKTYKCLQPHTSLAGWEPSNVPALWQLQGGHGGIRNNLNTENPLWDAIVGLGFLKDGVKNIPSFLSTDNIGTRETFLAGLIDSDGYVTDEHGIKATIKTIHTSVRDGLVSLARSLGLVVSVNAEPAKVDMNVTKHKISYAIYMSGGDVLLNVLSKCAGSKKFRPAPAAAFARECRGFYFELQELKEDDYYGITLSDDSDHQFLLGSQVVVQN**MDPETCPCPSGGSCTCADSCKCEGCKCTSCKKSCCSCCPAECEKCAKDCVCKGGEAAEAEAEKCSCCQ-**

IMPACT purification

**_1_MDPETCPCPSGGSCTCADSCKCEGCKCTSCKKSCCSCCPAECEKCAKDCVCKGGEAAEAEAEKCSCCQ_68_**-

**Figure S1**. Sequences of fusion construct and purified WT-MT3. The WT-MT3 was expressed as a fusion protein with Chitin Binding Domain and Intein. The WT-MT3 (without any additional residues) was purified by IMPACT purification.

The sequence of SUMO-MT3 fusion protein:

**M**GSSHHHHHHSTSDSEVNQEAKPEVKPEVKPETHINLKVSDGSSEIFFKIKKTTPLRRLMEAFAKRQGKEMDSLRFLYDGIRIQADQTPEDLDMEDNDIIEAHREQIGGS**MDPETCPCPSGGSCTCADSCKCEGCKCTSCKKSCCSCCPAECEKCAKDCVCKGGEAAEAEAEKCSCCQ-**

Ulp1

Ni^2+^-NTA purification

GSMDPETCPCPSGGSCTCADSCKCEGCKCTSCKKSCCSCCPAECEKCAKDCVCKGGEAAEAEAEKCSCCQ_70_**-**

**Figure S2**. Sequences of SUMO-MT3 fusion construct and purified GS-MT3. The GS-MT3 was expressed as a fusion protein with His_6_-SUMO. The fusion protein was purified by Ni^2+^-NTA and was then treated with Ulp1. The GS-MT3 (with extra Gly-Ser residues) was collected in flow through of the second Ni^2+^-NTA purification.

The sequence of SUMO-LPETG-MT3 fusion protein:

MGSSHHHHHHSTSDSEVNQEAKPEVKPEVKPETHINLKVSDGSSEIFFKIKKTTPLRRLMEAFAKRQGKEMDSLRFLYDGIRIQADQTPEDLDMEDNDIIEAHREQIGGS**LPETGMDPETCPCPSGGSCTCADSCKCEGCKCTSCKKSCCSCCPAECEKCAKDCVCKGGEAAEAEAEKCSCCQ-**

Ca^2+^, eSrtA

Ni^2+^-NTA purification

**GMDPETCPCPSGGSCTCADSCKCEGCKCTSCKKSCCSCCPAECEKCAKDCVCKGGEAAEAEAEKCSCCQ_69_-**

**Figure S3**. Sequences of SUMO-LPETG-MT3 fusion construct and purified G-MT3. The LPETG-MT3 was expressed as a fusion protein with His_6_-SUMO. The fusion protein was purified by Ni^2+^-NTA and was then treated with eSrtA. The G-MT3 (with extra Gly residue) was collected in flow through of the second Ni^2+^-NTA purification.

The sequence of SUMO-eSrtA-LPETG-MT3 fusion protein:

**MKHHHHHHPMSDYDIPTTENLYFQGAMGNDHINLKVAGQDGSVVQFKIKRHTPLSKLMKAYCERQGLSMRQIRFRFDGQPINETDTPAQLEMEDEDTIDVFQQQTGG**SMQAKPQIPKDKSKVAGYIEIPDADIKEPVYPGPATREQLNRGVSFAEENESLDDQNISIAGHTFIDRPNYQFTNLKAAKKGSMVYFKVGNETRKYKMTSIRNVKPTAVEVLDEQKGKDKQLTLITCDDYNEETGVWETRKIFVATEVKHM**LPETGMDPETCPCPSGGSCTCADSCKCEGCKCTSCKKSCCSCCPAECEKCAKDCVCKGGEAAEAEAEKCSCCQ-**

Ca^2+^

Ni^2+^-NTA purification

**G**MDPETCPCPSGGSCTCADSCKCEGCKCTSCKKSCCSCCPAECEKCAKDCVCKGGEAAEAEAEKCSCCQ_69_-

**Figure S4**. Sequences of eSrtA-MT3 fusion construct and purified G-MT3. This protein was expressed as a fusion protein having His_6_-SUMO-eSrtA-LPETG-MT3. The fusion protein was loaded on Ni^2+^-NTA resin and on-bead sortase-mediated auto-cleavage was induced by adding 5 mM CaCl_2_. The G-MT3 (with extra Gly residue) was collected in flow-through.

The sequence of His_6_-eSrtA-LPETG-MT3 fusion control protein (without SUMO):

MGSSHHHHHHSSGLVPRGSHMENLYFQGVEFLNCCPGCCMEPGSMQAKPQIPKDKSKVAGYIEIPDADIKEPVYPGPATREQLNRGVSFAEENESLDDQNISIAGHTFIDRPNYQFTNLKAAKKGSMVYFKVGNETRKYKMTSIRNVKPTAVEVLDEQKGKDKQLTLITCDDYNEETGVWETRKIFVATEVKHM**LPETGMDPETCPCPSGGSCTCADSCKCEGCKCTSCKKSCCSCCPAECEKCAKDCVCKGGEAAEAEAEKCSCCQ-**

**
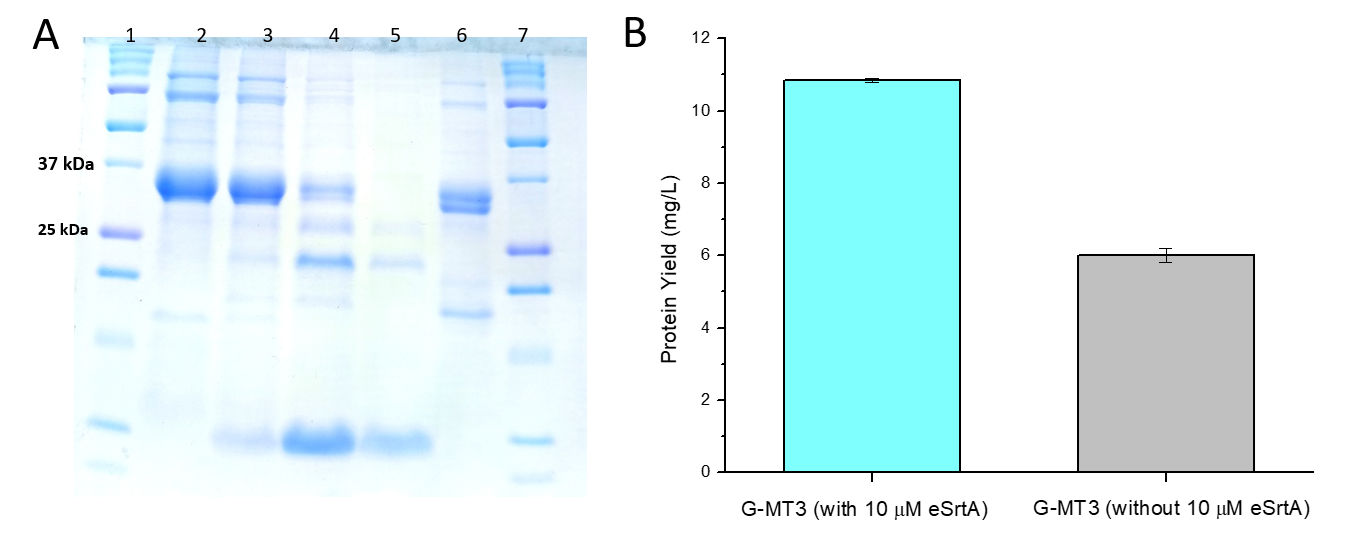
**

**Figure S5**. Inducible on-bead sortase-mediated cleavage of eSrtA-MT3. A) SDS-PAGE profile of on-bead sortase-mediated cleavage of eSrtA-MT3. Lane 1 = protein ladder, Lane 2 = fusion protein after binding to the Ni^2+^-NTA beads, Lane 3 = cleavage of eSrtA-MT3 in the absence of Ca^2+^ ion, Lane 4 = cleavage of eSrtA-MT3 in the presence of Ca^2+^ ion, Lane 5 = purified G-MT3, Lane 6 = uncleaved eSrtA-MT3 left on the Ni^2+^-NTA beads, and lane 7 = protein ladder. B) Comparison of total protein yield (mg/L) obtained during Ca^2+^ induced on-bead cleavage of the eSrtA-MT3 in the presence and absence of external 10 µM eSrtA.

**Figure S56**. SEC purification profile of apo-forms of WT-MT3, GS-MT3 (with additional Gly-Ser) and G-MT3 (with additional Gly) on a Superdex™ 75 Increase 10/300 GL column using 10 mM HCl, and absorbance was recorded at 220 and 280 nm. The retention volume for all apo-MTs was 19 mL.


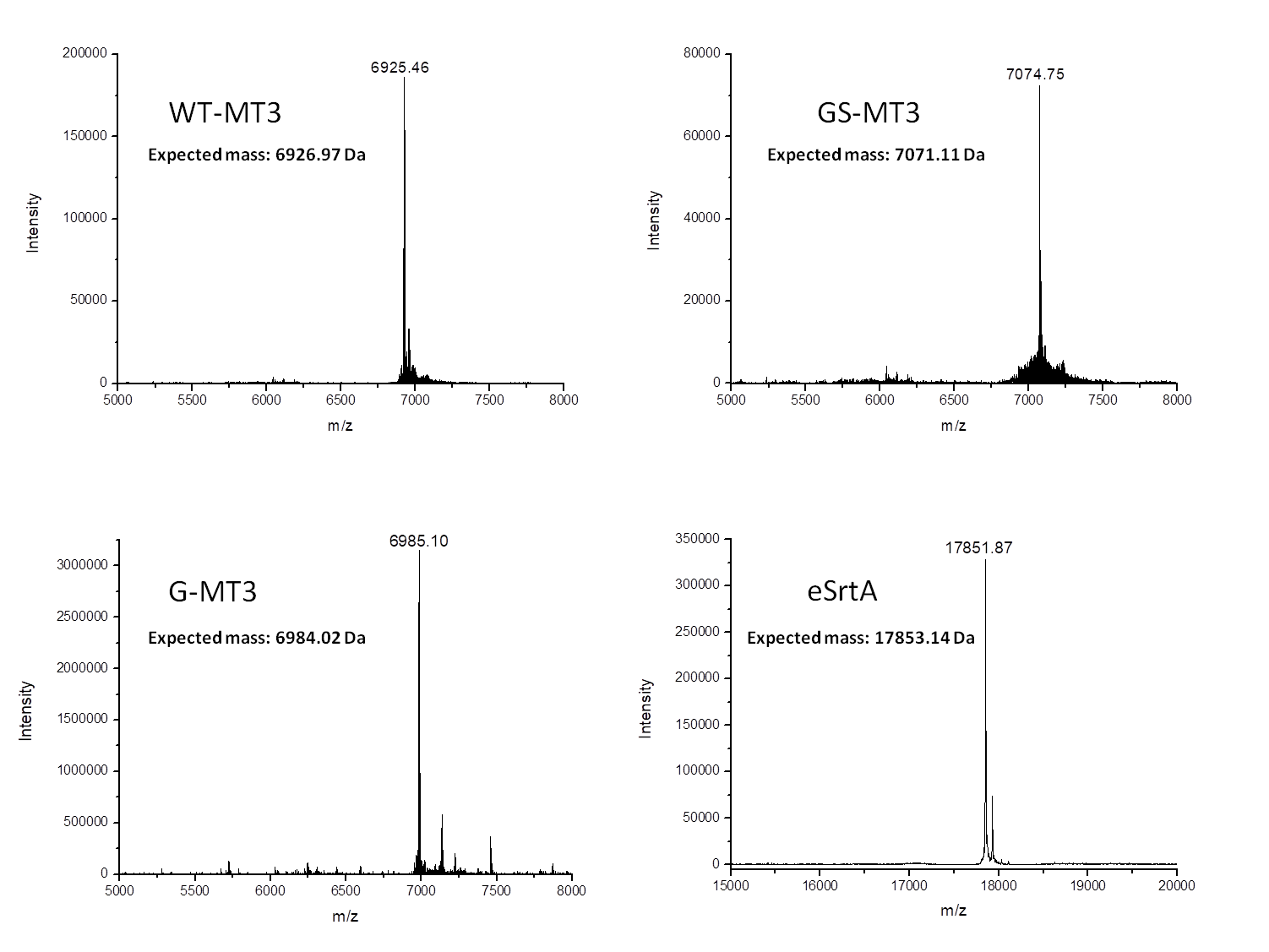


**Figure S7**. Deconvoluted ESI-MS spectra of WT-MT3, GS-MT3, G-MT3, and eSrtA obtained in this study.

**Figure S8**. UV-vis monitored Zn(II) titrations to 1 µM metal-free G-MT3, GS-MT3, and MT3-WT in 50 mM borate pH 7.4, 100 mM NaClO_4_, 100 µM TCEP.


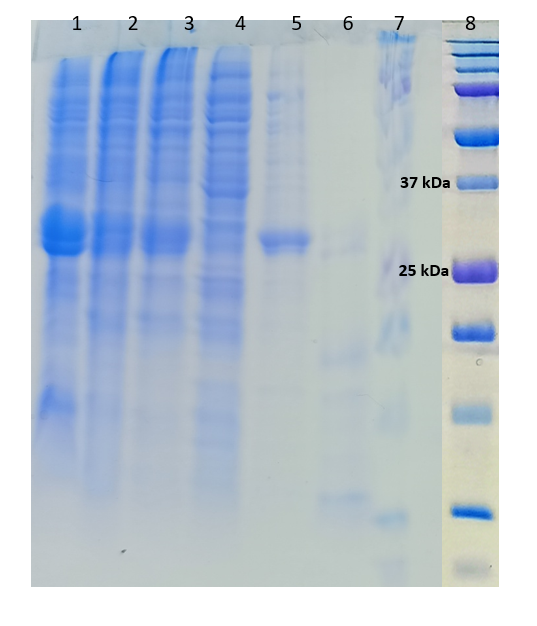


**Figure S9**. SDS-PAGE purification profile of His_6_-eSrtA-LPETG-MT3 (eSrtA-MT3 without SUMO). Lane 1 = pellet, Lane 2 = lysate, Lane 3 = supernatant, Lane 4 = flowthrough, Lane 5 = wash, Lane 6 = eluted G-MT3, and lane 7 = protein ladder. A large fraction of the expressed protein was found in the pellet fraction. Loss of fusion protein during the washing step (with 50 mM imidazole) was also seen. Lane 8: A reference protein ladder (the same ladder as in lane 7).

**Table S1**. Molecular masses and extinction coefficients of fusion constructs and purified proteins. Experimental masses were obtained from ESI-MS measurements.

| Peptide/protein sequence | No. of  aa  residues | Experimental mass average (Da) | Expected mass average  (Da) | Extinction coefficient  (M^-1^ cm^-1^)*^a^* |
| --- | --- | --- | --- | --- |
| CBD-Intein-MT3 | 593 | - | 65 342.44 | 65 780 |
| WT-MT3 | 68 | 6925.46 | 6 926.97 | - |
| SUMO-MT3 | 178 | - | 19 499.95 | 1 490 |
| GS-MT3 | 70 | 7074.75 | 7 071.10 | - |
| SUMO-LPETG-MT3 | 183 | - | 19 997.50 | 1 490 |
| SUMO-eSrtA-LPETG-MT3 | 331 | - | 36 897.76 | 18 910 |
| His_6_-eSrtA-LPETG-MT3 | 267 | *^b^* | 29 280.25 | 15 930 |
| G-MT3 | 69 | 6985.10 | 6 984.02 | - |
| eSrtA | 156 | 17851.87 | 17 853.14 | 14 440 |
| Ulp1 | 235 | - | 27 263.10 | 29 910 |

*^a^* Molar extinction coefficients were calculated using ExPASy ProtParam tool, https://web.expasy.org/cgi-bin/peptide_mass/peptide-mass.pl.

*^b^* For that construct molecular mass was not measured since it was used for expression control only.

**Table S2**. List of all the primers used in this study.

| 1. | SUMO-MT3 Fwd | **AAAAAAGGATCCATGGACCCGGAAACC** |
| --- | --- | --- |
| 2. | SUMO-MT3 Rev | **CCCCCCAAGCTTTTACTGACAGCAACTACACTTTTCAG** |
| 3. | LPETG-MT3 Fwd1 | **GGAAACCGGCATGGACCCGGAAACC** |
| 4. | LPETG-MT3 Fwd2 | **AAAAAAGGATCCCTGCCGGAAACCGGCATGGACC** |
| 5. | eSrtA BamHI Fwd | **CCCCCC GGATCCATGCAAGCTAAACCTCAAATTC** |
| 6. | eSrtA NdeI Rev | **CCCCCC CATATGTTTGACTTCTGTAGCTACAAAG** |
| 7. | MT3 NdeI Fwd | **AAAA CATATGCTTCCGGAAACCGGGATGGACCC** |
| 8. | MT3 XhoI Rev | **GGGG CTCGAG TTACTGACAGCAACTACACTTTTC** |
